# Supplementary material for: Hbo1 and Msl complexes preserve differential compaction and H3K27me3 marking of active and inactive X chromosomes during mitosis
Source: Nat Cell Biol. 2025 Sep 8;27(9):1482–95. doi: 10.1038/s41556-025-01748-0 (PMC12431858; doi:10.1038/s41556-025-01748-0)
Supplement: Supplementary file 1 — Gating strategy used for flow cytometry analyses. [file 41556_2025_1748_MOESM1_ESM.pdf]

nature cell biology

Supplementary information https://doi.org/10.1038/s41556-025-01748-0

Hbo1 and Msl complexes preserve differential compaction and H3K27me3 marking of active and inactive X chromosomes during mitosis

In the format provided by the authors and unedited

Gating strategy panels for the flow cytometry analyses

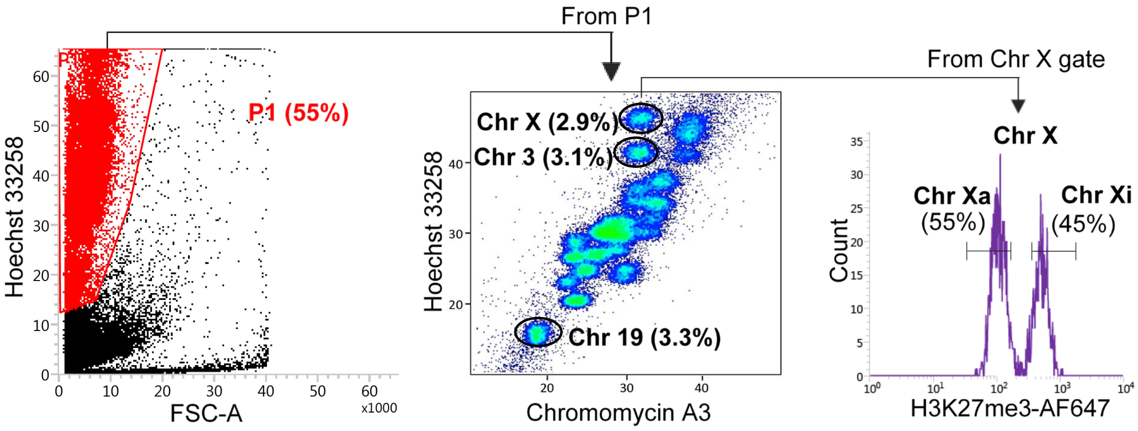

Example of gating strategy used for FACS sorting of metaphase chromosomes 3,19, Xi and Xa from female mpre-B cells is shown. Percentage of each gate is indicated. Chromosomes (P1) were first gated on Hoechst 33258 and Forward scatter FSC-A signal to gate out debris and clumps. P1 was then used to create a chromosome karyotype by plotting Hoechst 33258 vs Chromomycin A3 fluorescence. Chr X gate was used to create H3K27me3-AF647 profile to discriminate Xa (H3K27me3 low) and Xi (H3K27me3 high).

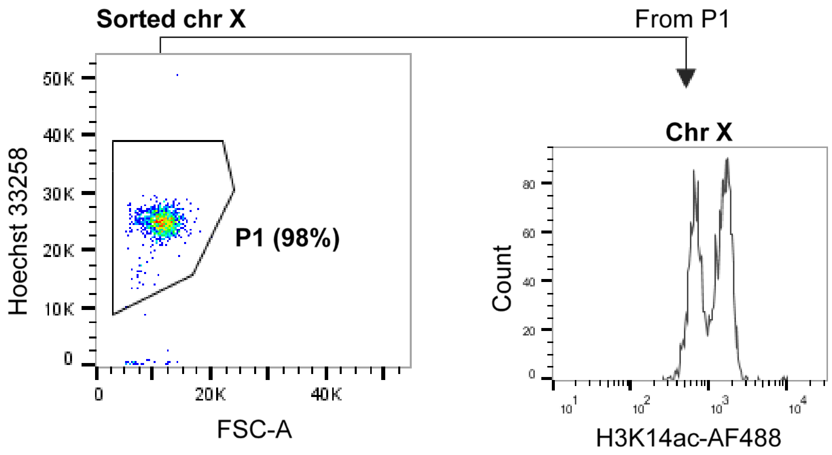

Example of gating strategy used for H3K14ac flow cytometry analysis on sorted chromosomes X. Percentage of each gate is indicated. Sorted Chr X (P1) was first gated on Hoechst 33258 and Forward scatter FSC-A signal. P1 gate was used to analyse H3K14ac profile.

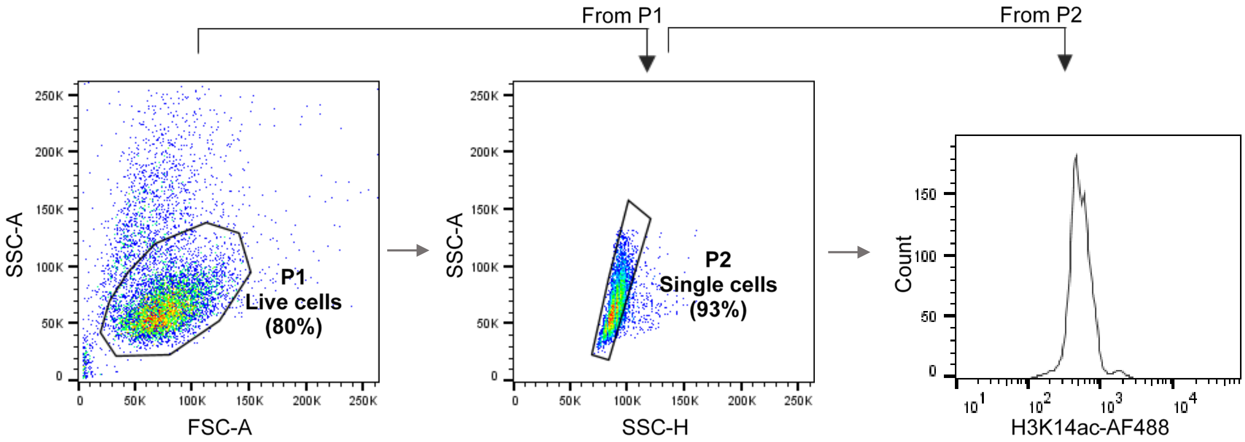

Example of gating strategy used for flow cytometry analysis of mpre-B cells immunostained with H3K14ac-AF488 antibody. Percentage of each gate is indicated. Cells were first gated based on forward scatter (FSC-A) and side scatter (SSC-A) and then gated based on side scatter height and area to exclude doublets. P2 (Single cells) gate was used to analyse H3K14ac profile.
