# Supplementary material for: Hbo1 and Msl complexes preserve differential compaction and H3K27me3 marking of active and inactive X chromosomes during mitosis
Source: Nat Cell Biol. 2025 Sep 8;27(9):1482–95. doi: 10.1038/s41556-025-01748-0 (PMC12431858; doi:10.1038/s41556-025-01748-0)
Supplement: Supplementary file 2 — Reporting Summary [file 41556_2025_1748_MOESM2_ESM.pdf]

nature portfolio

Corresponding author(s): Amanda G. Fisher
Dounia Djeghloul

Last updated by author(s): May 27, 2025

Reporting Summary

Nature Portfolio wishes to improve the reproducibility of the work that we publish. This form provides structure for consistency and transparency in reporting. For further information on Nature Portfolio policies, see our Editorial Policies and the Editorial Policy Checklist.

Statistics

For all statistical analyses, confirm that the following items are present in the figure legend, table legend, main text, or Methods section.

n/a	Confirmed	
☐	☒	The exact sample size (*n*) for each experimental group/condition, given as a discrete number and unit of measurement
☐	☒	A statement on whether measurements were taken from distinct samples or whether the same sample was measured repeatedly
☐	☒	The statistical test(s) used AND whether they are one- or two-sided<br>*Only common tests should be described solely by name; describe more complex techniques in the Methods section.*
☐	☒	A description of all covariates tested
☒	☐	A description of any assumptions or corrections, such as tests of normality and adjustment for multiple comparisons
☐	☒	A full description of the statistical parameters including central tendency (e.g. means) or other basic estimates (e.g. regression coefficient) AND variation (e.g. standard deviation) or associated estimates of uncertainty (e.g. confidence intervals)
☐	☒	For null hypothesis testing, the test statistic (e.g. *F*, *t*, *r*) with confidence intervals, effect sizes, degrees of freedom and *P* value noted<br>*Give P values as exact values whenever suitable.*
☒	☐	For Bayesian analysis, information on the choice of priors and Markov chain Monte Carlo settings
☒	☐	For hierarchical and complex designs, identification of the appropriate level for tests and full reporting of outcomes
☒	☐	Estimates of effect sizes (e.g. Cohen's *d*, Pearson's *r*), indicating how they were calculated

Our web collection on statistics for biologists contains articles on many of the points above.

Software and code

Policy information about availability of computer code

Data collection	BD FACS software (vl.2.0.142) and BD DIVA (v9.1) were used to collect flow cytometry data. Micro-Manager (v2.0, Olympus IX70 microscope) and LAS-AF (2.7.3.9723, SPS II microscope) were used to collect imaging data. Quantitative real-time PCR data was collected using Bio-Rad CFX Manager software (v3.1).
Data analysis	Flow cytometry data was analysed using FlowJo software (v10.8.1). lmageJ/Fiji (version 1.54e) was used for image analysis including mitotic chromosome size measurements. Proteomic data was analysed using the Label-Free Quantification algorithm in the MaxQuant software platform (v1.6.10.43). The Perseus software (vl.6.7.0 and v1.6.15.0) was used for both statistical analysis and data visualisation of the proteomics data. Gene Ontology analysis was performed at http://geneontology.org/.

For manuscripts utilizing custom algorithms or software that are central to the research but not yet described in published literature, software must be made available to editors and reviewers. We strongly encourage code deposition in a community repository (e.g. GitHub). See the Nature Portfolio guidelines for submitting code & software for further information.

Data

Policy information about availability of data

All manuscripts must include a data availability statement. This statement should provide the following information, where applicable:

- Accession codes, unique identifiers, or web links for publicly available datasets
- A description of any restrictions on data availability
- For clinical datasets or third party data, please ensure that the statement adheres to our policy

The mass spectrometry proteomics data have been deposited to the ProteomeXchange Consortium via the PRIDE partner repository with the dataset identifier PXD054014. Gene Ontology annotations for use in Perseus were downloaded from http://annotations.perseus-framework.org (mainAnnot.mus_musculus.txt). All other relevant data supporting the key findings of this study are available within the article and Supplementary Tables provided in the submission.

Research involving human participants, their data, or biological material

Policy information about studies with human participants or human data. See also policy information about sex, gender (identity/presentation), and sexual orientation and race, ethnicity and racism.

Reporting on sex and gender	*Use the terms sex (biological attribute) and gender (shaped by social and cultural circumstances) carefully in order to avoid confusing both terms. Indicate if findings apply to only one sex or gender; describe whether sex and gender were considered in study design; whether sex and/or gender was determined based on self-reporting or assigned and methods used. Provide in the source data disaggregated sex and gender data, where this information has been collected, and if consent has been obtained for sharing of individual-level data; provide overall numbers in this Reporting Summary. Please state if this information has not been collected. Report sex- and gender-based analyses where performed, justify reasons for lack of sex- and gender-based analysis.*
Reporting on race, ethnicity, or other socially relevant groupings	*Please specify the socially constructed or socially relevant categorization variable(s) used in your manuscript and explain why they were used. Please note that such variables should not be used as proxies for other socially constructed/relevant variables (for example, race or ethnicity should not be used as a proxy for socioeconomic status). Provide clear definitions of the relevant terms used, how they were provided (by the participants/respondents, the researchers, or third parties), and the method(s) used to classify people into the different categories (e.g. self-report, census or administrative data, social media data, etc.) Please provide details about how you controlled for confounding variables in your analyses.*
Population characteristics	*Describe the covariate-relevant population characteristics of the human research participants (e.g. age, genotypic information, past and current diagnosis and treatment categories). If you filled out the behavioural & social sciences study design questions and have nothing to add here, write "See above."*
Recruitment	*Describe how participants were recruited. Outline any potential self-selection bias or other biases that may be present and how these are likely to impact results.*
Ethics oversight	*Identify the organization(s) that approved the study protocol.*

Note that full information on the approval of the study protocol must also be provided in the manuscript.

Field-specific reporting

Please select the one below that is the best fit for your research. If you are not sure, read the appropriate sections before making your selection.

☒ Life sciences ☐ Behavioural & social sciences ☐ Ecological, evolutionary & environmental sciences

For a reference copy of the document with all sections, see nature.com/documents/nr-reporting-summary-flat.pdf

Life sciences study design

All studies must disclose on these points even when the disclosure is negative.

Sample size	A minimum of three independent biological replicates (n = 3) was used for experiments in this study, including for proteomics, flow cytometry, FISH, chromosome size and imaging analysis. The data were highly consistent between the biological replicates. Chromosome size and fluorescence intensity measurements were based on at least 85 chromosomes for pre-B cells and a minimum of 25 chromosomes for primary fibroblasts (Mof inducible KO and Msl2 KO), collected over three independent experiments.
Data exclusions	There was no exclusion/inclusion of samples in the analysis.
Replication	Proteomic experiments were conducted with at least three biological replicates. Flow cytometry and chromosome imaging analyses were also performed using a minimum of three biological replicates to ensure reproducibility of key findings. qRT-PCR analyses were carried out in three independent biological replicates, each with technical triplicates to account for pipetting errors.
Randomization	Randomization was not relevant to this study as there was no assignment of samples to different experimental groups.

Blinding | Blinding was not relevant since there was no assignment of samples to different experimental groups.

Reporting for specific materials, systems and methods

We require information from authors about some types of materials, experimental systems and methods used in many studies. Here, indicate whether each material, system or method listed is relevant to your study. If you are not sure if a list item applies to your research, read the appropriate section before selecting a response.

Materials & experimental systems

n/a	Involved in the study
☐	☒ Antibodies
☐	☒ Eukaryotic cell lines
☒	☐ Palaeontology and archaeology
☒	☐ Animals and other organisms
☒	☐ Clinical data
☒	☐ Dual use research of concern
☒	☐ Plants

Methods

n/a	Involved in the study
☒	☐ ChIP-seq
☐	☒ Flow cytometry
☒	☐ MRI-based neuroimaging

Antibodies

Antibodies used

Primary antibodies used were: H3K27me3-AF647 (clone:C36B11, 12158S, Cell Signalling), macroH2A1 (ab37264, Abcam), H4K16ac-AF488 (clone: E2B8W, 56999, Cell Signalling), H3K14ac-AF488 (clone: EP964Y, ab277918, Abcam), H4ac-AF488 (acetyl K5 + K8 + K12 + K16) (clone: EPR16606, ab223995, Abcam), Trim28 (ab10484, Abcam, lot:GR288493-50), Kat7 (ab70183, Abcam, lot:GR3364428-11), Noc2L (PA5-101730, Invitrogen), H3K14ac (clone: EP964Y, ab52946, Abcam), H4K16ac (ab109463, Abcam), H4ac (06-598, Merck Millipore, lot: 3473490). Dilutions used for the antibodies are provided in the manuscript. Secondary antibodies used were: goat anti-rabbit Alexa Fluor 568, A-11011, Invitrogen, goat anti-rabbit Alexa 488, A11034, Invitrogen.

Validation

H3K27me3-AF647 (clone:C36B11, 12158, Cell Signalling), wide species reactivity expected including mouse, tested for use in flow cytometry and ICC/IF on supplier's website.
MacroH2A1 (ab37264, Abcam), reacts with human and mouse samples, tested for use in ICC/IF and knockout-validated on supplier's website.
H4K16ac-AF488 (clone: E2B8W, 56999, Cell Signalling), wide species reactivity expected including mouse, tested for use in flow cytometry and ICC/IF on supplier's website. Validated in this study for flow cytometry using conditional Mof-KO mouse fibroblasts.
H3K14ac-AF488 (clone: EP964Y, ab277918, Abcam), the unconjugated version of this antibody (same clone) reacts with mouse, rat and human, tested on supplier's website for several applications including chIP and ICC/IF and predicted for use in flow cytometry. Validated in this study for flow cytometry using mouse pre-B cells treated with Kat7 histone acetyltransferase inhibitor.
H4ac-AF488 (acetyl K5 + K8 + K12 + K16) (clone: EPR16606, ab223995, Abcam), the unconjugated version of this antibody (same clone) reacts with mouse, rat and human samples, validated on supplier's website for ICC/IF.
Trim28 (ab10484, Abcam), reacts with mouse and human samples, validated for use in IHC on supplier's website.
Kat7 (ab70183, Abcam), reacts with mouse, rat and human samples and validated for use in ICC/IF on supplier's website.
Noc2L (PA5-101730, Invitrogen), reacts with mouse, rat and human samples and validated for ICC/IF on supplier's website.
H3K14ac (clone: EP964Y, ab52946, Abcam), reacts with mouse, rat and human, tested on supplier's website for several applications including chIP and ICC/IF.
H4K16ac (ab109463, Abcam), reacts with mouse, rat and human samples and validated for ICC/IF on supplier's website.
H4ac (06-598, Merck Millipore), wide species reactivity predicted and tested for ICC/IF on supplier's website.

Eukaryotic cell lines

Policy information about cell lines and Sex and Gender in Research

Cell line source(s)

Abelson-transformed pre-B cell lines were previously generated in our lab (Lavagnolli et.al, Genes Dev 2015). Mouse Embryonic Fibroblasts (MEFs) used in this study were Mof fl/fl Caag ERT2-cre MEFs and Msl2 KO or WT MEFs, from Asifa Akhtar's lab (Sheikh et al, Oncogene 2016; Sun et al, Nature 2023).

Authentication

All cell lines were tested for karyotype and genotyped using appropriate primers.

Mycoplasma contamination

All cell lines were tested negative for mycoplasma contamination.

Commonly misidentified lines
(See ICLAC register)

No commonly misidentified lines were used in this study

Plants

Seed stocks

Report on the source of all seed stocks or other plant material used. If applicable, state the seed stock centre and catalogue number. If plant specimens were collected from the field, describe the collection location, date and sampling procedures.

Novel plant genotypes

Describe the methods by which all novel plant genotypes were produced. This includes those generated by transgenic approaches, gene editing, chemical/radiation-based mutagenesis and hybridization. For transgenic lines, describe the transformation method, the number of independent lines analyzed and the generation upon which experiments were performed. For gene-edited lines, describe the editor used, the endogenous sequence targeted for editing, the targeting guide RNA sequence (if applicable) and how the editor was applied.

Authentication

Describe any authentication procedures for each seed stock used or novel genotype generated. Describe any experiments used to assess the effect of a mutation and, where applicable, how potential secondary effects (e.g. second site T-DNA insertions, mosiacism, off-target gene editing) were examined.

Flow Cytometry

Plots

Confirm that:

☒ The axis labels state the marker and fluorochrome used (e.g. CD4-FITC).

☒ The axis scales are clearly visible. Include numbers along axes only for bottom left plot of group (a 'group' is an analysis of identical markers).

☐ All plots are contour plots with outliers or pseudocolor plots.

☒ A numerical value for number of cells or percentage (with statistics) is provided.

Methodology

Sample preparation

Chromosome sorting: Chromosomes were extracted from pre-B cell lines or MEFs and stained with Hoechst 33258 and Chromomycin A3. Individual chromosome populations were purified using BD Influx with BD FACS software (v1.2.0.142). Hoechst 33258 was excited using a 355 nm laser (350mW) and fluorescence was collected using a 400 nm long pass filter in combination with a 500 nm short pass filter. Chromomycin A3 was excited using a 457 nm laser (300mW) and fluorescence was collected using a 500 nm long pass filter in combination with a 600 nm short pass filter. For isolating Xi and Xa, H3K27me3-AF647 was excited using a 637 nm laser (160 mW), and the resulting fluorescence was collected using a 660/30 bandpass filter. Forward scatter was measured using a 488 nm laser (200 mW). Chromosomes were sorted using a 70 µm nozzle tip, with a drop drive frequency of ~96 kHz and sheath pressure at 65 PSI and were collected into FACS tubes containing polyamine buffer.
Intracellular staining: For intracellular staining of acetylated histones, cells were fixed and permeabilised using the BD Cytofix/Cytoperm Fixation/Permeabilization Kit according to manufacturer's instructions. Cells were then incubated with conjugated antibodies diluted in permeabilisation buffer for 1 h at RT. After washes, cells were resuspended in FACS buffer (PBS containing 2% FBS) for analysis.

Instrument

Chromosomes: BD Influx equipped with spatially separated air cooled lasers. Intracellular staining: BD FACSymphony flow cytometer.

Software

BD FACS software (vl.2.0.142, Influx); BD DIVA (v9.1, Symphony)

Cell population abundance

Gating strategy used for sorting chromosome X (Xi or Xa), 19, 3 is provided in the manuscript. Sort purity of individual chromosomes was assessed by DNA-FISH with mouse chromosome-specific paints. 80-95% sort purity was achieved.

Gating strategy

Chromosomes were first gated on a plot of high Hoechst 33258 vs low Forward scatter signal to gate out debris and clumps. This first gate was then used to create a chromosome karyotype by plotting Hoechst 33258 vs Chromomycin A3 fluorescence. For analysis of cells, gates and voltage were set by first running unstained samples. Cells were first gated based on forward scatter and side scatter and then gated based on side scatter height and area to exclude doublets.
Gating strategies used for chromosome sorting/analysis as well as histone acetylation staining in cells are provided in the Supplementary Information File.

☒ Tick this box to confirm that a figure exemplifying the gating strategy is provided in the Supplementary Information.
