# Supplementary material for: Hbo1 and Msl complexes preserve differential compaction and H3K27me3 marking of active and inactive X chromosomes during mitosis
Source: Nat Cell Biol. 2025 Sep 8;27(9):1482–95. doi: 10.1038/s41556-025-01748-0 (PMC12431858; doi:10.1038/s41556-025-01748-0)
Supplement: Supplementary file 6 — Unprocessed blots. [file 41556_2025_1748_MOESM6_ESM.pdf]

# Uncropped gel images

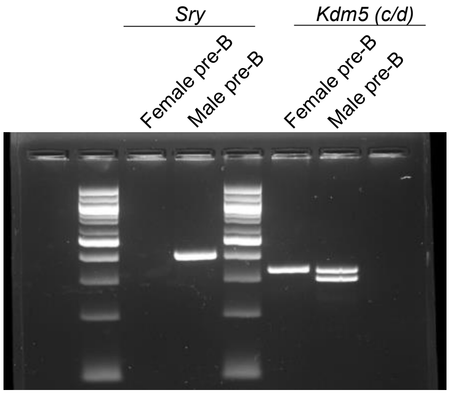


Uncropped gel image: Source data for Extended data Fig. 1a

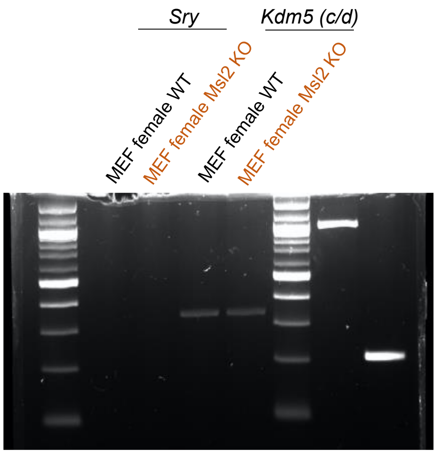


Uncropped gel image: Source data for Extended data Fig. 5a

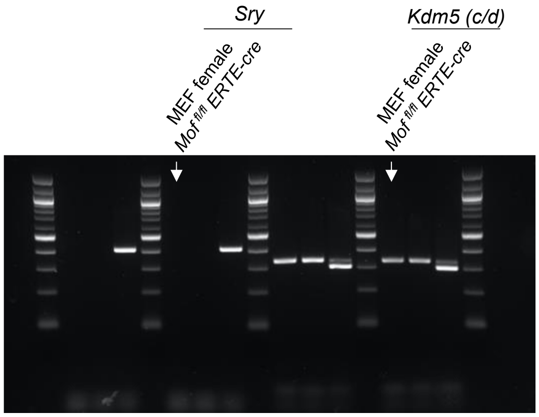


Uncropped gel image: Source data for Extended data Fig. 6a
